# The dose–response relationship between working hours and prevalence of hypertension in construction workers: evidence from Wuhan, China

**DOI:** 10.3389/fpubh.2025.1693554

**Published:** 2025-12-11

**Authors:** Xuyu Chen, Lishan Zhu, Zhiliang Chen, Yuehui Liang, Yang Yang, Xiaodong Tan, Zhicheng Jiang

**Affiliations:** 1 Health Management Center, The Second Affiliated Hospital, Jiangxi Medical College, Nanchang University, Nanchang, China; 2 Health Inspection Department, Wuhan Prevention and Treatment Center for Occupational Diseases, Wuhan, China; 3 Women’s Hospital School of Medicine, Zhejiang University, Chongqing, China; 4 Chongqing Qijiang District Center for Disease Control and Prevention, Chongqing, China; 5 School of Public Health, Wuhan University, Wuhan, China

**Keywords:** hypertension, construction workers, dose–response relationship, restricted cubic spline, occupational health

## Abstract

**Background:**

The rapid urban expansion and renewal in China have increased the workload and work intensity for construction workers. Prolonged work hours prevent these builders from having adequate recovery time, which may be associated with higher susceptibility to occupational injuries and chronic diseases. This study aimed to explore the dose–response relationship between weekly working hours and hypertension among construction workers.

**Methods:**

A cross-sectional study was carried out from June to July 2022 in Wuhan, China. A stratified cluster random sampling method was used, and all construction workers at their respective construction sites were invited to participate. Unconditional logistic regression and restricted cubic spline (RCS) models were utilized to evaluate the dose–response relationship between weekly working hours and hypertension. Subgroup and sensitivity analyses were conducted to assess the robustness of the results.

**Results:**

A total of 750 construction workers were included in our study, and the prevalence of hypertension among them was 18.00%. Workers with hypertension reported longer weekly working hours (67.64 ± 12.70 h/week) than those without hypertension (59.72 ± 12.81 h/week). The likelihood of having hypertension was found to be 4.22 (95% *CI* = 2.10–8.49) times higher for those in the Q3 group and 4.90 (95% *CI* = 2.26–10.64) times higher (66–70 h/week) for those in the Q4 group (71–140 h/week) compared to the Q1 group (10–54 h/week). The RCS analysis showed a non-linear dose–response relationship between weekly working hours and hypertension (*P*_non-linear_< 0.05), and the results were consistent across subgroups.

**Conclusion:**

In this cross-sectional study, longer weekly working hours were associated with higher odds of hypertension among construction workers.

## Introduction

The construction industry is a cornerstone of global urbanization, yet its workforce, construction workers, face persistent challenges from high-intensity physical labor, irregular schedules, and multiple occupational hazards (e.g., noise and dust) (1, 2). Recently, rapid urban expansion and renewal in China have led to an unprecedented demand for construction. Consequently, construction workers face escalating work intensity and growing workloads, with overtime, shift rotations, and night shifts becoming increasingly common. Extended working hours and sustained physical exhaustion leave workers in a state of fatigue with insufficient time for recovery, heightening their susceptibility to occupational injuries (3). Epidemiological evidence reveals that this population has a significantly higher prevalence of chronic diseases than the general public (4, 5). Hypertension, a major risk factor for chronic conditions such as cardiovascular disease, has emerged as a critical health concern. Factors such as body mass index (BMI), the use of biomass for cooking, tobacco use, dietary patterns, leisure-time physical activity, and access to health facilities have been identified as significant contributors to hypertension (6). Notably, the World Health Organization highlights occupational stress and behavioral patterns as key contributors to hypertension (7, 8), while construction workers’ chronic overwork potentially amplifies their risks of developing hypertension.

Current epidemiological evidence on the relationship between working hours and hypertension is contradictory. While some studies report positive linear correlations in the total sample or specific subgroups (9, 10), others have found no significant association (11, 12). A study from the United States even indicates that increased working hours reduce the risk of hypertension (13). The majority of these previous studies primarily concentrate on quantifying the strength of the association while overlooking the shape of the dose–response relationship. A Japanese study revealed a U-shaped association between working hours and the prevalence of diabetes (14), suggesting that the relationship between working hours and health outcomes may not be linear. The methodological approaches in this field have predominantly relied on logistic regression models—an approach that inherently limits the detection of non-linear relationships. The restricted cubic spline (RCS) model integrated with generalized linear models has emerged as a robust analytical framework for identifying complex non-linear associations across biomedical research domains.

In 2021, the total number of workers engaged in the construction industry in China was approximately 55.58 million (15). With accelerated urbanization, the health of workers in this labor-intensive sector has attracted increasing attention. Research on hypertension in this population is crucial for developing interventions that can reduce the associated disease burden, which has substantial implications for public health. Although previous studies have suggested potential associations between working hours and hypertension risk, insufficient evidence persists regarding dose–response relationships specific to construction workers. Therefore, this study aimed to explore the dose–response relationship between the prevalence of hypertension and working hours among construction workers in Wuhan, providing a scientific basis for targeted health interventions.

## Methods

### Study design and setting

A cross-sectional study was conducted in Wuhan, China, from June to July 2022, using a stratified cluster random sampling method. As a major metropolitan area and a key economic hub in central China, Wuhan is undergoing rapid urbanization. The pressures and working conditions faced by the construction workforce in this setting are likely representative of those in many other large cities across China. The sampling process first involved stratifying all districts into three economic tiers: high-tier (GDP > 10 billion yuan), medium-tier (6–10 billion yuan), and low-tier (<6 billion yuan). Construction sites were then proportionally selected from each stratum based on the total number of sites registered with the Wuhan Prevention and Treatment Center for Occupational Diseases (Wuhan PTCOD). This proportionally stratified sampling approach aimed to improve the socioeconomic representativeness of our sample. All on-site construction workers who met the inclusion criteria were included in the final analysis.

### Participants

The sample size was calculated using the following formula for prevalence studies: $N=\text{deff}\frac{{u_{1-\alpha /2}}^{2}p(1-p)}{d^{2}}$ . A design effect (*deff*) of 2 was applied. The calculation assumed a 95% confidence level (two-sided), corresponding to a ‘u’ value of 1.96, a hypertension prevalence (p) of 27.5% (16), and an allowable error (d) of 0.05. Considering a potential 10% non-response rate, the total required sample size was calculated to be 674 participants. The inclusion criteria were as follows: (1) age: ≥18 years, (2) at least 1 year of experience in the construction industry, (3) voluntary participation with informed consent, and (4) the ability to complete questionnaires under researcher guidance. The exclusion criteria were as follows: (1) a diagnosis of mental illness or experiencing significant adverse life events within the preceding 6 months, (2) having severe systemic diseases affecting major organs (e.g., cardiac, cerebral, pulmonary, hepatic, or renal) or hematologic dysfunction. During data collection, additional exclusion criteria were applied to workers lacking either physical examination records or blood samples, along with those with incomplete questionnaire responses. A total of 36 participants were ultimately excluded (Supplementary Figure 1), yielding an inclusion rate of 93.28% (750/804) for the final analysis.

### Measurements

A self-designed questionnaire was used to collect essential demographic information, detailed lifestyle patterns, and occupation-specific characteristics of construction workers, including age, gender, education level, smoking status (active and passive), alcohol consumption, physical exercise habits, salt intake, comorbidity, sleep status, weekly working hours, work shifts, and occupational exposure. Weight, height, waist circumference (WC), hip circumference (HC), and blood pressure (BP) levels were measured by certified nurses, following standardized protocols during the physical examination. After a seated rest of at least 5 min, BP was measured twice on the right arm using a calibrated automatic electronic blood pressure monitor. The two measurements were taken at least 1–2 min apart, and the average of the two readings was used for all analyses. Following an 8-h overnight fast, 5 mL of peripheral venous blood was drawn in the morning. The samples were transported to the Wuhan PTCOD Laboratory Department for biochemical analysis, which included assessments of liver and kidney function, blood glucose levels, lipid profiles, and a complete blood count.

### Outcomes and operational definitions

Hypertension is defined by any of the following three criteria: (1) an average systolic BP of ≥ 140 mmHg or diastolic BP of ≥90 mmHg, (2) a self-reported diagnosis of hypertension by a physician, or (3) current use of antihypertensive medication (17). Operational definitions included the following: (1) active smoking: smoking at least one cigarette per day for a minimum of 6 months; (2) passive smoking: exposure to secondhand tobacco smoke for at least 15 min on 1 or more days per week; (3) alcohol consumption: drinking alcohol at least once per week for a minimum of 6 months, regardless of the quantity; (4) physical exercise: engagement in moderate-intensity exercise for at least 20 min per session, with a frequency of no less than once per week; (5) comorbidity: the presence of at least one chronic condition in addition to hypertension; (6) sleep status: this is measured by the Pittsburgh Sleep Quality Index (PSQI) scores (1–21), where higher scores represent poorer sleep quality; (7) work shifts: work schedules that extend beyond conventional daytime hours (18); (8) BMI: this is calculated as weight (kg) divided by the square of height (m); and (9) waist-to-hip ratio (WHR): this is calculated as WC (cm) divided by HC (cm).

### Quality control

The project investigator coordinated with site managers to schedule the surveys and ensured that workers fasted before blood collection to guarantee accurate results. Investigators were trained to conduct face-to-face interviews using a standardized questionnaire with clear instructions. Quality control officers reviewed all completed questionnaires to identify and rectify any missing data. Data entry and verification were performed independently by two staff members to promptly identify and correct any errors.

### Statistical analysis

Continuous variables with normal and non-normal distributions are presented as mean ± standard deviation and median (interquartile range), respectively. Categorical variables are summarized as frequencies and percentages. Comparisons between the hypertensive and non-hypertensive groups were performed using Pearson’s chi-squared test (categorical variables), Student’s *t*-test (normally distributed continuous variables), and Mann–Whitney *U*-test (non-normally distributed continuous variables). Variable selection was performed using univariate analysis followed by the least absolute shrinkage and selection operator (LASSO) regression. A multivariable logistic regression analysis was performed to assess the independent association between weekly working hours and hypertension. Linear trend testing was conducted by transforming weekly working hours into within-group medians. The dose–response relationship between weekly working hours and hypertension risk was evaluated using a restricted cubic spline (RCS) model. The robustness of the primary findings was assessed using subgroup and sensitivity analyses. LASSO regression and RCS analyses were conducted using *R 4.1.2*, while all other statistical analyses were performed using *SPSS 26.0*. A two-sided *p*-value of < 0.05 was considered statistically significant.

## Results

### Participant characteristics and univariate analysis

A total of 750 construction workers were included in the study, with a hypertension prevalence of 18.00%. The sample comprised 644 men (85.87%) and 106 women (14.13%), with a mean age of 47.01 ± 10.93 years. The majority of participants (45.33%) attained a junior high school education. Additionally,24.27% reported a preference for a salty diet, and 26.00% had at least one chronic comorbidity other than hypertension. The mean BMI, WC, and WHR were 24.40 ± 3.86 kg/m^2^, 85.69 ± 10.05 cm, and 0.89 ± 0.07, respectively. Notably, construction workers with hypertension reported longer weekly working hours (67.64 ± 12.70 h/week) than those without hypertension (59.72 ± 12.81 h/week). Detailed characteristics of constuction workers are shown in Table 1, and non-significant biochemical indicators are presented in Supplementary Table 1.

**Table 1:** Description of participants and group comparison between construction workers with/without hypertension.

Group	All	Without hypertension (*n* = 615)	With hypertension (***n*** = 135)	Statistic	*p*
Age (years, mean ± SD)	47.01 ± 10.93	45.94 ± 10.96	51.87 ± 9.42	5.826^#^	<0.001^***^
Age (%)					
≤40	198 (26.40)	181 (91.41)	17 (8.59)	31.609	<0.001^***^
41–50	185 (24.67)	160 (86.49)	25 (13.51)		
51–60	329 (43.87)	250 (75.99)	79 (24.01)		
≥61	38 (5.07)	24 (63.16)	14 (36.84)		
Gender (%)					
Men	644 (85.87)	529 (82.14)	115 (17.86)	0.063	0.802
Women	106 (14.13)	86 (81.13)	20 (18.87)		
Educational level (%)					
Primary school and below	200 (26.67)	165 (82.50)	35 (17.50)	9.146	0.027^*^
Middle school	340 (45.33)	278 (81.76)	62 (18.24)		
High school or technical secondary school	132 (17.60)	100 (75.76)	32 (24.24)		
Junior college or above	78 (10.40)	72 (92.31)	6 (7.69)		
BMI (kg/m^2^,mean ± SD)	24.40 ± 3.86	24.14 ± 3.83	25.61 ± 3.77	4.076^#^	<0.001^***^
BMI levels (%)					
<18.5	29 (3.87)	27 (93.10)	2 (6.90)	21.749	<0.001^***^
18.5–23.9	343 (45.73)	301 (87.76)	42 (12.24)		
24.0–27.9	266 (35.47)	207 (77.82)	59 (22.18)		
≥28.0	112 (14.93)	80 (71.43)	32 (28.57)		
WC (cm, mean ± SD)	85.69 ± 10.05	84.57 ± 9.54	90.80 ± 10.75	6.710	<0.001^***^
WHR (mean ± SD)	0.89 ± 0.07	0.88 ± 0.07	0.91 ± 0.06	4.858	<0.001^***^
Active smoking (%)					
No	380 (50.67)	338 (88.95)	42 (11.05)	25.188	<0.001^***^
Yes	370 (49.33)	277 (74.86)	93 (25.14)		
Passive smoking (%)					
No	345 (46.00)	276 (80.00)	69 (20.00)	1.731	0.188
Yes	405 (54.00)	339 (83.70)	66 (16.30)		
Alcohol consumption (%)					
No	422 (56.27)	341 (80.81)	81 (19.19)	0.933	0.334
Yes	328 (43.73)	274 (83.54)	54 (16.46)		
Physical exercise (%)					
No	626 (83.47)	516 (82.43)	110 (17.57)	0.470	0.493
Yes	124 (16.53)	99 (79.84)	25 (20.16)		
Sleep status [median, (IQR)]	4.00 [3.00–7.00]	4.00 [3.00–7.00]	4.00 [3.00–6.00]	−2.330^$^	0.020^*^
Salty diet					
Yes	182 (24.27)	103 (56.59)	79 (43.41)	105.097	<0.001^***^
No	568 (75.73)	512 (90.14)	56 (9.86)		
Comorbid diseases (%)					
No	555 (74.00)	487 (87.75)	68 (12.25)	47.778	<0.001^***^
Yes	195 (26.00)	128 (65.64)	67 (34.36)		
Working time (hours/week, mean ± SD)	61.15 ± 13.14	59.72 ± 12.81	67.64 ± 12.70	6.512^#^	<0.001^***^
Work in shifts (%)					
No	624 (83.20)	520 (83.33)	104 (16.67)	4.474	0.034^*^
Yes	126 (16.80)	95 (75.40)	31 (24.60)		
Occupational exposure (%)					
Dust	194 (25.87)	163 (84.02)	31 (15.98)	18.791	<0.001^***^
Noise	106 (14.13)	93 (87.74)	13 (12.26)		
Dust and noise	133 (17.73)	92 (69.17)	41 (30.83)		
Others	317 (42.27)	267 (84.23)	50 (15.77)		
Occupation (%)					
Bricklayer	71 (9.47)	57 (80.28)	14 (19.72)	3.362	0.762
Carpenter	131 (17.47)	112 (85.50)	19 (14.50)		
Mechanical equipment operator/driver	67 (8.93)	54 (80.60)	13 (19.40)		
Steel bender	59 (7.87)	48 (81.36)	11 (18.64)		
Scaffolder	53 (7.07)	46 (86.79)	7 (13.21)		
Handyman	82 (10.93)	69 (84.15)	13 (15.85)		
Others	287 (38.27)	229 (79.79)	58 (20.21)		
γ-GTP [U/L, median (IQR)]	23.20 [15.80, 37.05]	22.05 [15.58, 35.48]	28.80 [20.40, 44.60]	−3.761^$^	<0.001^***^
GLU [mmol/L, median (IQR)]	5.00 [4.69, 5.51]	4.97 [4.65, 5.43]	5.25 [4.90, 6.13]	−5.036^$^	<0.001^***^
TG [mmol/L, median (IQR)]	1.63 [1.12,2.44]	1.59 [1.11, 2.38]	1.80 [1.19,3.00]	−2.317^$^	0.021^*^
HDL-C (mmol/L, mean ± SD)	1.26 ± 0.30	1.27 ± 0.31	1.21 ± 0.25	−2.116^#^	0.035^*^
PLT (10^9/L, mean ± SD)	219.59 ± 52.62	221.75 ± 52.95	209.72 ± 50.13	−2.414^#^	0.016*
MCHC (g/L, mean ± SD)	334.47 ± 8.42	334.14 ± 8.55	335.93 ± 7.64	2.243^#^	0.025^*^
PDW (fL, mean ± SD)	16.16 ± 0.36	16.14 ± 0.36	16.26 ± 0.32	3.305^#^	0.001^**^

^#^ and ^$^ in the statistics indicate the use of a t-test and a Mann-Whitney U-test for group comparisons. The group comparison without marks on the statistics is the Chi-square test. ^*^ indicates *p* < 0.05, ^**^ indicates *p* < 0.01, and ^***^ indicates *p* < 0.001.

### Variable selection

In the univariate analysis, hypertension status was significantly associated with multiple variables (all *p* < 0.05), including age, educational level, BMI, WC, WHR, active smoking, sleep status, salty diet, comorbidities, weekly working hours, work shifts, occupational exposure, and various laboratory parameters such as γ-GTP, GLU, TG, PLT, MCHC, and PDW (Table 1). Using LASSO regression analysis (*λ.1se* = 0.0391), six potential predictors were identified: age, WC, active smoking, salty diet, comorbidities, and weekly working hours.

### Trend test and non-linearity test

Collinearity diagnostics showed that VIFs were all < 6, indicating no significant multicollinearity among the included variables. Additionally, no significant interactions were identified between pairs of categorical variables (Supplementary Table 2). Weekly working hours were categorized into quartiles (Q1–Q4), with Q1 (10–54 h/week) as the reference group. We built three sequential models (models 1, 2, and 3) to calculate the odds ratios (ORs) for hypertension across the quartiles of working hours. As displayed in Table 2, the ORs progressively increased from Q1 to Q4. Compared to Q1, Q3 (66–70 h/week) and Q4 (71–140 h/week) were associated with higher odds of hypertension (*p* < 0.05). A significant trend was observed (*P* for trend < 0.001), and a significant non-linear relationship was detected (*P* for non-linear = 0.010).

**Table 2:** Relationship between weekly working hours and risk of hypertension and the trend test.

Model^$^	Group	within-group median	Hypertension cases	OR (95%*CI*)	*p* ^#^	*P* for trend
Model 1	Q_1_ [10–54]	32.0	20			
	Q_2_ [55–63]	58.5	36	1.43 (0.81–2.59)	0.224	
	Q_3_ [64–70]	66.5	45	3.58 (2.04–6.46)	<0.001^***^	
	Q_4_ [71–140]	105.0	34	6.00 (3.23–11.41)	<0.001^***^	<0.001^***^
Model 2	Q_1_ [10–54]	32.0	20			
	Q_2_ [55–63]	58.5	36	1.43 (0.80–2.60)	0.236	
	Q_3_ [64–70]	66.5	45	3.52 (1.99–6.43)	<0.001^***^	
	Q_4_ [71–140]	105.0	34	6.31 (3.34–12.22)	<0.001^***^	<0.001^***^
Model 3	Q_1_ [10–54]	32.0	20			
	Q_2_ [55–63]	58.5	36	1.30 (0.67–2.61)	0.442	
	Q_3_ [64–70]	66.5	45	4.18 (2.12–8.54)	<0.001^***^	
	Q_4_ [71–140]	105.0	34	4.83 (2.26–10.59)	<0.001^***^	<0.001^***^

^$^All the above models use unconditional logistic regression. Model 1 is unadjusted for variables, Model 2 is adjusted for age, and Model 3 is adjusted for age, active smoking, WC, a salty diet, and comorbidity. ^#^is the *p*-value for the risk of hypertension.^***^ indicates *p* < 0.001.

### Dose–response relationship analysis

**Figure 1:**
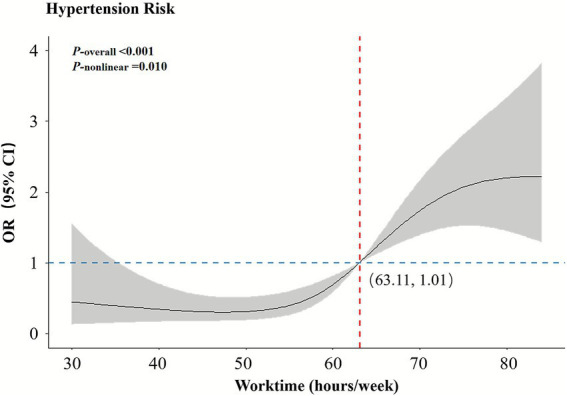
Dose–response relationship between weekly working hours and hypertension.

**Figure 2:**
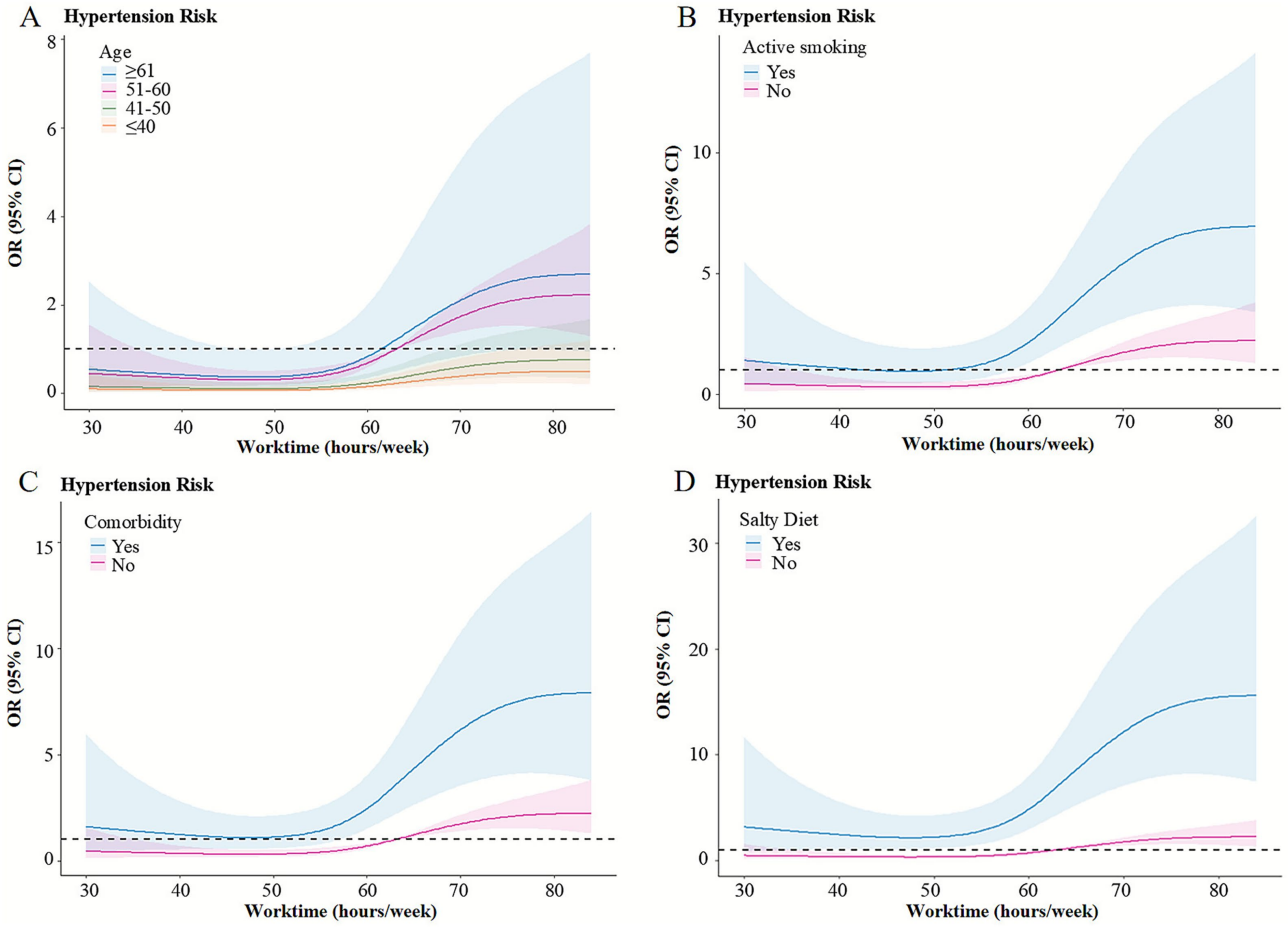
Subgroup analyses of the dose–response relationship between weekly working hours and hypertension risk. **(A)** subgroup for different age **(B)** subgroup for active smoking **(C)** subgroup for comorbidity **(D)** subgroup for salty diet.

The model achieved an optimal fit with four knots (AIC = 485.84, BIC = 536.66). After adjusting for age, WC, active smoking, salty diet, and comorbidity, the association between weekly working hours and hypertension among construction workers exhibited a non-linear increase, as illustrated in Figure 1. Using 63 h/week as the reference (OR = 1), working hours below this threshold (e.g., 40 and 56 h/week) were associated with lower odds of hypertension (OR < 1), whereas working hours above this level (e.g., 84 h/week) were associated with higher odds of hypertension (OR > 1). The relationship between weekly working hours and hypertension displayed an “S”-shaped curve, with the lowest risk occurring at approximately 47 h/week (*OR* = 0.30, 95% *CI* = 0.18–0.51), after which the risk began to increase. Subgroup analyses revealed that these dose–response trends were largely consistent across different populations, supporting the robustness of the primary findings (Figure 2).

### Sensitivity analysis

**Figure 3:**
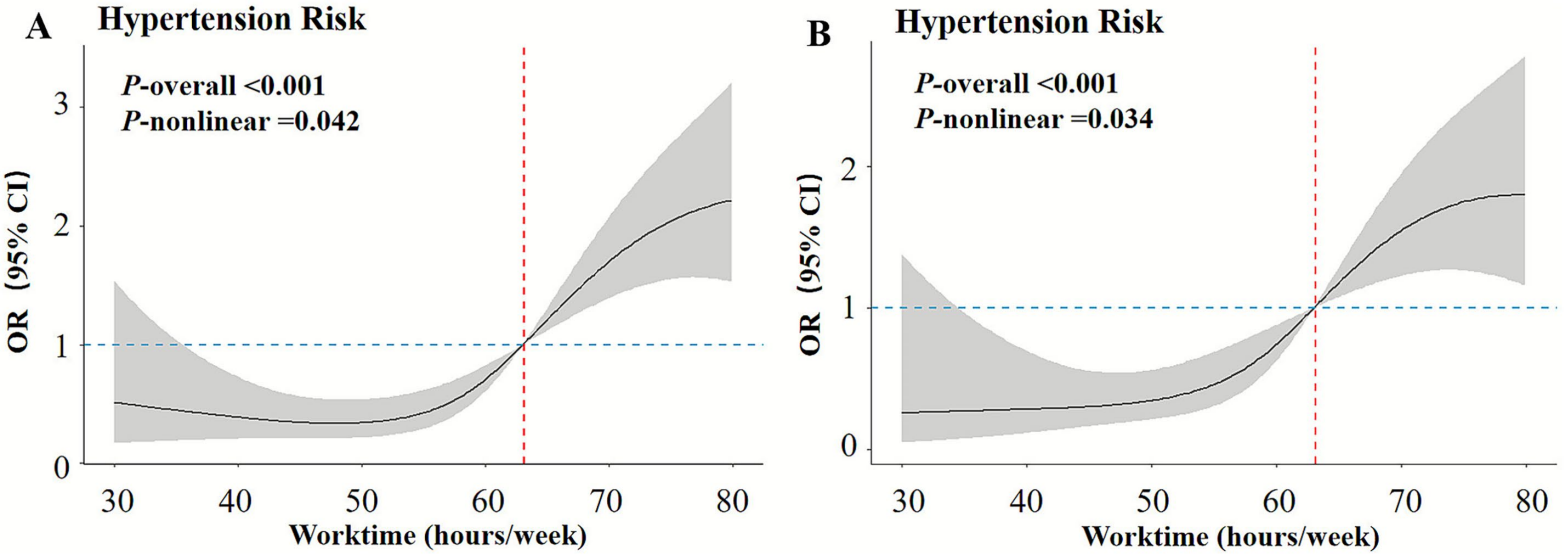
Sensitivity analysis. **(A)** Excluding workers engaged in shift work and **(B)** after the exclusion of workers exposed to occupational noise.

When excluding workers engaged in shift work, the *p*-value for non-linearity was 0.042, with an AIC of 476.56 and a BIC of 507.61. Compared to Q1, the ORs for Q2, Q3, and Q4 were 1.26 (0.62–2.60), 4.01 (1.96–8.51), and 4.40 (1.74–11.24), respectively. Similarly, after the exclusion of workers exposed to occupational noise, the *p*-value for non-linearity was 0.034, with an AIC of 360.07 and a BIC of 389.73. The corresponding ORs for Q2, Q3, and Q4 were 2.67 (1.01–7.74), 12.70 (4.71–38.73), and 10.36 (3.55–33.37), respectively. In both sensitivity analyses, the dose–response relationship between weekly working hours and hypertension maintained a consistent S-shaped curve (Figures 3A,B), indicating the stability of the observed non-linear association.

## Discussion

Contrary to expectations, construction workers exhibited a lower prevalence of hypertension (18.00%) compared to the general population of Chinese adults (27.5%) (16), despite their chronic exposure to extended shifts, hazardous environments, and unhealthy lifestyles and diets. This discrepancy may be explained by two synergistic protective mechanisms: (1) the healthy-worker effect, the healthy-worker selection bias favoring physically fit individuals during recruitment, and (2) occupationally sustained high-intensity activity, which aligns with hypertension prevention guidelines. Empirical studies confirm an inverse relationship between physical activity levels and systolic/diastolic pressure levels (19). Despite the lower overall prevalence, hypertensive construction workers may face heightened occupational vulnerability. Evidence suggests that poorly controlled hypertension is associated with an increased risk of occupational injuries.

Our study found that construction workers in Wuhan worked an average of 61.15 h per week, which is substantially above the Chinese legal limit of 44 h (20). Furthermore, weekly working hours were significantly longer among hypertensive workers compared to non-hypertensive workers. In this cross-sectional study, workers in the Q3 and Q4 groups had significantly higher odds of hypertension than those in the Q1 group. This positive association is consistent with findings from other countries, including the United States (9), Ethiopia (21), and Korea (22). Prolonged workplace exposure may increase contact with both physical (e.g., dust, noise, and toxic chemicals) and psychosocial (e.g., work stress, social isolation, and high demands) hazards. Furthermore, long working hours have been linked to adverse health behaviors such as smoking, alcohol use, and obesity (10, 23), all of which are established risk factors for hypertension.

Another key finding of our study was the non-linear relationship between weekly working hours and hypertension. Many previous studies have not fully captured the dynamic trend across the continuum of working hours (9, 10). Our application of the restricted cubic spline (RCS) model effectively addressed this methodological gap. Direct evidence for the dose–response relationship is limited. One notable study by Cheng et al. reported a U-shaped association between working hours and hypertension in a general population of 12,080 Chinese adults (24). Several factors may explain the discrepancy between our S-shaped curve and the U-shaped association reported by Cheng et al.: first, the study populations were fundamentally different, as our study exclusively focused on construction workers, who are exposed to distinct behavioral lifestyles and occupational environments compared to the general population studied by Cheng et al. Second, the pronounced male predominance in our sample (male-to-female ratio of 6.08:1 vs. 1.06:1) is noteworthy, given the established correlation between hypertension and gender (25, 26). Finally, differences in the number and placement of knots in the RCS models can also influence the shape of the dose–response curve.

Within the current health and regulatory systems in China, access to hypertension care for construction workers remains limited. Although occupational safety laws and public health programs offer some level of screening and education, many migrant and informal laborers lack access to comprehensive medical insurance. To address this gap, collaborative efforts between health authorities and employers should focus on establishing regular on-site blood pressure screenings and facilitating referrals to primary care. Furthermore, strengthening regulations to enforce rest periods, overtime limits, and compulsory health insurance for all workers would help mitigate hypertension risk factors and improve treatment access.

However, our study is not devoid of limitations. First, the cross-sectional design precludes causal inference, and the observed associations should be interpreted as correlational rather than causal. Second, our sample was drawn exclusively from construction sites in Wuhan. While Wuhan is a major urban center in China, regional variations in economic development, work practices, and workforce demographics indicate that our findings may not be fully generalizable to all construction workers across China. Therefore, the results should be interpreted with caution in broader contexts. Third, the definition of hypertension was based on the average of two BP measurements taken during a single visit, a self-reported diagnosis, or medication use. While this approach is pragmatic for large-scale field surveys, it does not adhere to the clinical guidelines that recommend multiple measurements over separate visits and may lead to an overestimation of the true prevalence. Fourth, the measurement of weekly working hours was based on self-reports, which can be affected by recall bias and social desirability bias. Finally, factors such as dietary habits, stress levels, and genetic predisposition were not fully accounted for, which may have influenced the observed non-linear relationship.

To address these issues, future studies should adopt longitudinal or interventional designs to establish causality and explore the effects of specific health interventions. Expanding the research to multiple provinces and incorporating more objective health metrics, such as detailed biomedical measurements and environmental assessments, would improve the generalizability and provide a stronger evidence base for policy formulation. Despite these limitations, our study provides robust evidence of a non-linear relationship between hypertension and weekly working hours among construction workers. These findings could guide the organization of work schedules for construction workers and hold certain public health significance.

## Conclusion

A non-linear dose–response relationship between weekly working hours and hypertension was observed among construction workers in Wuhan. While awaiting longitudinal validation, policymakers may consider extending statutory health insurance to all on-site workers, enforcing mandatory rest periods and overtime limits, and establishing structured on-site blood pressure screenings with established referral pathways to primary care. Further validation in multi-regional studies is recommended before these findings can be generalized to inform national occupational health policies.

## Data Availability

The raw data supporting the conclusions of this article will be made available by the authors, without undue reservation.

## Supplementary material

SUPPLEMENTARY FIGURE 1 The flaw diagram of sample screening.

SUPPLEMENTARY TABLE 1 Description of participant biochemical analysis and group comparison between construction workers with/without hypertension.

SUPPLEMENTARY TABLE 2 Interaction test between each pair of independent variables.
