# Supplementary material for: The dose–response relationship between working hours and prevalence of hypertension in construction workers: evidence from Wuhan, China
Source: Front Public Health. 2025 Dec 11;13:1693554. doi: 10.3389/fpubh.2025.1693554 (PMC12738893; doi:10.3389/fpubh.2025.1693554)
Supplement: SUPPLEMENTARY TABLE 1 — Description of participant biochemical analysis and group comparison between construction workers with/without hypertension. [file Table_1.DOCX]

**Table 1.** Description of participants and group comparison between construction workers with/without hypertension

| **Group** | **All** | **Without hypertension**  **(n=615)** | **With hypertension**  **(n=135)** | **Statistic** | ***P*** |
| --- | --- | --- | --- | --- | --- |
| Age(years,mean±SD) | 47.01±10.93 | 45.94±10.96 | 51.87±9.42 | 5.826^#^ | <0.001^***^ |
| Age(%) |  |  |  |  |  |
| ≤40 | 198 (26.40) | 181 (91.41) | 17 (8.59) | 31.609 | <0.001^***^ |
| 41-50 | 185 (24.67) | 160 (86.49) | 25 (13.51) |  |  |
| 51-60 | 329 (43.87) | 250 (75.99) | 79 (24.01) |  |  |
| ≥61 | 38 (5.07) | 24 (63.16) | 14 (36.84) |  |  |
| Gender(%) |  |  |  |  |  |
| male | 644 (85.87) | 529 (82.14) | 115 (17.86) | 0.063 | 0.802 |
| female | 106 (14.13) | 86 (81.13) | 20 (18.87) |  |  |
| Educational level(%) |  |  |  |  |  |
| primary school and below | 200 (26.67) | 165 (82.50) | 35 (17.50) | 9.146 | 0.027^*^ |
| middle school | 340 (45.33) | 278 (81.76) | 62 (18.24) |  |  |
| high school or technical secondary school | 132 (17.60) | 100 (75.76) | 32 (24.24) |  |  |
| junior college or above | 78 (10.40) | 72 (92.31) | 6 (7.69) |  |  |
| BMI(kg/m2,mean±SD） | 24.40±3.86 | 24.14±3.83 | 25.61±3.77 | 4.076^#^ | <0.001^***^ |
| BMI degree(%） |  |  |  |  |  |
| ＜18.5 | 29 (3.87) | 27 (93.10) | 2 (6.90) | 21.749 | <0.001^***^ |
| 18.5-23.9 | 343 (45.73) | 301 (87.76) | 42 (12.24) |  |  |
| 24.0-27.9 | 266 (35.47) | 207 (77.82) | 59 (22.18) |  |  |
| ≥28.0 | 112 (14.93) | 80 (71.43) | 32 (28.57) |  |  |
| WC(cm,mean±SD) | 85.69±10.05 | 84.57±9.54 | 90.80±10.75 | 6.710 | <0.001^***^ |
| WHR(mean±SD) | 0.89±0.07 | 0.88±0.07 | 0.91±0.06 | 4.858 | <0.001^***^ |
| Active smoking(%) |  |  |  |  |  |
| no | 380 (50.67) | 338 (88.95) | 42 (11.05) | 25.188 | <0.001^***^ |
| yes | 370 (49.33) | 277 (74.86) | 93 (25.14) |  |  |
| Passive smoking(%) |  |  |  |  |  |
| no | 345 (46.00) | 276 (80.00) | 69 (20.00) | 1.731 | 0.188 |
| yes | 405 (54.00) | 339 (83.70) | 66 (16.30) |  |  |
| Alcohol drinking(%) |  |  |  |  |  |
| no | 422 (56.27) | 341 (80.81) | 81 (19.19) | 0.933 | 0.334 |
| yes | 328 (43.73) | 274 (83.54) | 54 (16.46) |  |  |
| Physical exercise(%) |  |  |  |  |  |
| no | 626 (83.47) | 516 (82.43) | 110 (17.57) | 0.470 | 0.493 |
| yes | 124 (16.53) | 99 (79.84) | 25 (20.16) |  |  |
| Sleep status[median, (IQR)] | 4.00[3.00-7.00] | 4.00[3.00-7.00] | 4.00[3.00-6.00] | -2.330^$^ | 0.020^*^ |
| Salty diet |  |  |  |  |  |
| yes | 182 (24.27) | 103 (56.59) | 79 (43.41) | 105.097 | <0.001^***^ |
| no | 568 (75.73) | 512 (90.14) | 56 (9.86) |  |  |
| Comorbidity diseases(%) |  |  |  |  |  |
| no | 555 (74.00) | 487 (87.75) | 68 (12.25) | 47.778 | <0.001^***^ |
| yes | 195 (26.00) | 128 (65.64) | 67 (34.36) |  |  |
| Working time( hours/week, mean±SD) | 61.15±13.14 | 59.72±12.81 | 67.64±12.70 | 6.512^#^ | <0.001^***^ |
| Work in shifts(%) |  |  |  |  |  |
| no | 624 (83.20) | 520 (83.33) | 104 (16.67) | 4.474 | 0.034^*^ |
| yes | 126 (16.80) | 95 (75.40) | 31 (24.60) |  |  |
| Occupational exposure(%) |  |  |  |  |  |
| dust | 194 (25.87) | 163 (84.02) | 31 (15.98) | 18.791 | <0.001^***^ |
| noise | 106 (14.13) | 93 (87.74) | 13 (12.26) |  |  |
| Dust and noise | 133 (17.73) | 92 (69.17) | 41 (30.83) |  |  |
| others | 317 (42.27) | 267 (84.23) | 50 (15.77) |  |  |
| Occupational type(%) |  |  |  |  |  |
| bricklayer | 71 (9.47) | 57 (80.28) | 14 (19.72) | 3.362 | 0.762 |
| carpenters | 131 (17.47) | 112 (85.50) | 19 (14.50) |  |  |
| mechanical equipment operator/driver | 67 (8.93) | 54 (80.60) | 13 (19.40) |  |  |
| steel bender | 59 (7.87) | 48 (81.36) | 11 (18.64) |  |  |
| scaffolder | 53 (7.07) | 46 (86.79) | 7（13.21) |  |  |
| handyman | 82 (10.93) | 69 (84.15) | 13（15.85) |  |  |
| others | 287 (38.27) | 229 (79.79) | 58（20.21) |  |  |
| γ-GTP[U/L,median(IQR)] | 23.20[15.80,37.05] | 22.05[15.58,35.48] | 28.80[20.40,44.60] | -3.761^$^ | <0.001^***^ |
| GLU[mmol/L,median(IQR)] | 5.00[4.69,5.51] | 4.97[4.65,5.43] | 5.25[4.90,6.13] | -5.036^$^ | <0.001^***^ |
| TG[mmol/L,median(IQR)] | 1.63[1.12,2.44] | 1.59[1.11,2.38] | 1.80[1.19,3.00] | -2.317^$^ | 0.021^*^ |
| HDL-C(mmol/L,mean±SD) | 1.26±0.30 | 1.27±0.31 | 1.21±0.25 | -2.116^#^ | 0.035^*^ |
| PLT(10^9/L,mean±SD) | 219.59±52.62 | 221.75±52.95 | 209.72±50.13 | -2.414^#^ | 0.016* |
| MCHC(g/L,mean±SD) | 334.47±8.42 | 334.14±8.55 | 335.93±7.64 | 2.243^#^ | 0.025^*^ |
| PDW(fL,mean±SD) | 16.16±0.36 | 16.14±0.36 | 16.26±0.32 | 3.305^#^ | 0.001^**^ |

Note: ^#^ and ^$^ in the statistic indicate the use of *t* test and *Mann-Whitney U* test for group comparison; The group comparison without marks on the statistic is the *Chi-square* test. ^*^ represents *P*<0.05, ^**^ represents *P*<0.01, ^***^ represents *P*<0.001.

**Table 2. Relationship between weekly working hours and risk of hypertension and Trend test**

| **Model^$^** | **Group** | **within-group median** | **Hypertension cases** | **OR (95%*CI*)** | ***P*^#^** | ***P* for trend** |
| --- | --- | --- | --- | --- | --- | --- |
| Model 1 | Q_1_ [10-54] | 32.0 | 20 |  |  |  |
|  | Q_2_ [55-63] | 58.5 | 36 | 1.43 (0.81-2.59) | 0.224 |  |
|  | Q_3_ [64-70] | 66.5 | 45 | 3.58 (2.04-6.46) | <0.001^***^ |  |
|  | Q_4_ [71-140] | 105.0 | 34 | 6.00 (3.23-11.41) | <0.001^***^ | <0.001^***^ |
| Model 2 | Q_1_ [10-54] | 32.0 | 20 |  |  |  |
|  | Q_2_ [55-63] | 58.5 | 36 | 1.43 (0.80-2.60) | 0.236 |  |
|  | Q_3_ [64-70] | 66.5 | 45 | 3.52 (1.99-6.43) | <0.001^***^ |  |
|  | Q_4_ [71-140] | 105.0 | 34 | 6.31 (3.34-12.22) | <0.001^***^ | <0.001^***^ |
| Model 3 | Q_1_ [10-54] | 32.0 | 20 |  |  |  |
|  | Q_2_ [55-63] | 58.5 | 36 | 1.30 (0.67-2.61) | 0.442 |  |
|  | Q_3_ [64-70] | 66.5 | 45 | 4.18 (2.12-8.54) | <0.001^***^ |  |
|  | Q_4_ [71-140] | 105.0 | 34 | 4.83 (2.26-10.59) | <0.001^***^ | <0.001^***^ |

Notes: ^$^All the above models using unconditional logistic regression. Model 1 is unadjusted for variables, Model 2 is adjusted for age, and Model 3 is adjusted for age, active smoking, WC, salty diet and comorbidity. ^#^is the *P*-value for the risk of hypertension.^***^ represents *P*<0.001.
